# Predictors and implications of medication non-adherence among psychiatric patients in Oman: A cross-sectional study

**DOI:** 10.5339/qmj.2025.98

**Published:** 2025-11-03

**Authors:** Salim Al-Huseini, Mohammed Qutishat, Mandhar Almaqbali, Mohammed Albalushi, Maryam Almashrafi, Khalid Alrisi, Mohamed Albreiki

**Affiliations:** 1 Department of Psychiatry, Al Masarra Hospital, Ministry of Health, Muscat, Sultanate of Oman; 2 Birmingham and Solihull Mental Health NHS Foundation Trust, Birmingham, UK; 3 Community and Mental Health Department, College of Nursing, Sultan Qaboos University, Muscat, Sultanate of Oman; 4 Department of Psychiatry, Sohar Hospital, Sohar, Sultanate of Oman; 5 Head of Psychiatry Emergency Department, Al Masarra Hospital, Ministry of Health, Muscat, Sultanate of Oman; 6 Department of Psychiatry, Sur Polyclinic, Sur, Sultanate of Oman *Email: Mohammed Qutishat mohqut@squ.edu.com

**Keywords:** Medication adherence, psychotropic medication, mental health, Oman

## Abstract

**Background:**

Mental health disorders significantly impact individual well-being and place a substantial burden on healthcare systems worldwide. In Oman, enhancing medication adherence among patients with psychiatric disorders is crucial, as non-compliance can worsen symptoms and lead to increased hospitalizations. This study aims to identify the predictors of medication non-adherence among patients at a tertiary care facility in Oman.

**Methods:**

A cross-sectional study was conducted at Al Masarra Hospital involving adult patients undergoing follow-up care for documented mental disorders. The Medication Adherence Rating Scale was used to assess medication adherence, while demographic and medical history data were collected through self-report. Descriptive statistics and linear regression were used to analyze the relationship between adherence and various factors.

**Results:**

Among the 151 participants, primarily aged between 26 and 45 years, the average medication adherence score was 5.71 (with a standard deviation of 2.45). Key predictors of non-adherence included marital status and treatment satisfaction, with unmarried individuals showing lower adherence levels. Interestingly, greater insight into one’s illness correlated with lower adherence, whereas higher treatment satisfaction was associated with better adherence.

**Conclusion:**

Medication non-adherence remains a significant challenge among psychiatric patients in Oman, influenced by psychosocial factors such as marital status, level of insight, and treatment satisfaction. Targeted interventions that address patient insight and enhance treatment engagement are essential for improving medication adherence and clinical outcomes.

## 1. BACKGROUND

Mental health has become a critical global concern, as millions of individuals struggle with various psychiatric disorders that can severely affect their quality of life.^1^ Conditions such as depression, anxiety, bipolar disorder, and schizophrenia not only undermine personal well-being but also impose substantial economic burdens on healthcare systems.^2,3^ The World Health Organization (WHO) estimates that one in four individuals will be affected by a mental health disorder at some point in their lives, highlighting the urgent need for effective treatments and interventions.^4,5^ Pharmacological treatment for mental health disorders has evolved significantly over the years, resulting in a wide range of medications designed to alleviate symptoms and improve patients’ overall functioning.^6,7^ Despite advancements in medication development, medication adherence remains a major challenge in determining patient health outcomes.^8^ Non-compliance with prescribed pharmacotherapy can worsen symptoms, increase hospitalizations, and place a higher overall burden on healthcare systems.^9^ Specifically, for individuals with mental health disorders, inadequate adherence to treatment can result in exacerbation of psychiatric symptoms, potentially leading to crises that require emergency intervention or inpatient care.^10^ This impacts the affected individual’s well-being and places additional burden on families and communities that may be unprepared to support those in distress.^11^ Ultimately, non-compliance can hinder recovery, perpetuating a cycle of mental health deterioration and escalating healthcare costs.

Non-compliance with prescribed pharmacotherapy in mental health can result from a lack of understanding of the medication’s importance, discouraging side effects, and limited access to healthcare resources.^12,13^ Furthermore, the stigma associated with mental health may prevent individuals from seeking help or discussing their treatment openly.^14^ Lastly, personal factors like inadequate social support and co-occurring substance use may further hinder adherence to prescribed treatment regimens.^15,16^

Research in Oman indicates suboptimal medication adherence across various mental health conditions, with studies on depression particularly highlighting this issue.^17^ Key predictors include diagnosis (with lower adherence in schizophrenia), illness duration, level of insight, cohabitation with family (a protective factor), and the presence of physical comorbidities.^17,18^ Mental health stigma—manifesting as fear of judgment, discrimination, and internalized shame—also significantly impairs adherence.^19–21^ Considering Oman’s unique socio-cultural context, understanding these factors is crucial for developing effective interventions and improving outcomes. The aim of this study is to identify the predictors of medication adherence among individuals with mental disorders in Oman.

## 2. METHODS

### 2.1. Study design, duration, and sitting

This cross-sectional study was conducted over a six-month period, from January to July 2024, at Al Masarra Hospital in Muscat, Oman. Al Masarra is a tertiary mental health facility that provides inpatient and outpatient services, as well as 24/7 emergency care, and accepts referrals from across the country.

### 2.2. Data collection process

Data were collected using a structured questionnaire, which participants completed in the outpatient waiting area prior to their scheduled appointments. The questionnaire collected demographic and clinical information, including participants’ age, gender, education level, and duration of treatment. The Medication Adherence Rating Scale (MARS) was used as the sole self-reported measure of adherence. Trained research assistants were available to assist the participants with any questions and to ensure confidentiality. When necessary, clinical data were cross-verified with the patient’s medical records.

### 2.3. Sampling and sample size calculation

Participants were recruited using a convenience sampling method, and the study included all patients over 18 years of age who were receiving follow-up care at the psychiatry clinic and had a documented mental disorder. Patients who declined consent or had incomplete records were excluded from the study. To determine the minimum sample size required to detect the effects of a specific intervention at a designated significance level, calculations were performed using G* Power version 3.1, with an effect size of 0.5 and a P-value error of 0.05. Accordingly, the study required a sample size of 180 patients.

### 2.4. Study Instruments

The study used the Medication Adherence Rating Scale (MARS) as the sole self-reported measure of medication adherence. Demographic data—including participants’ age, gender, education level, and treatment duration—were collected using a structured questionnaire to identify the potential predictors and describe the sample.

#### 2.4.1. Medication adherence rating scale (MARS)

The Medication Adherence Rating Scale (MARS) was developed by Thompson and colleagues in 2000 to assess how well individuals with mental health conditions—especially those with schizophrenia—adhere to their prescribed medication. Originally developed in English, the scale has demonstrated good reliability and validity, with an internal reliability score of 0.75. The MARS consists of ten questions, each scored as either “No = 0” or “Yes = 1”. The level of adherence is determined by adding the scores, with the total score ranging from 0 (indicating poor adherence) to 10 (indicating good adherence). A higher total score indicates better medication adherence. The tool was translated into Arabic and back-translated to ensure accuracy. The reliability test among our participants yielded a Cronbach’s α of 0.835.

### 2.5. Data analysis

The statistical analyses were performed using IBM SPSS Statistics for Windows, Version 26 (SPSS Inc., Chicago, IL, USA). A descriptive statistical analysis was conducted to summarize the sample’s demographic characteristics and medication history, including frequency percentages, means, and standard deviations (SDs). Linear regression analysis was conducted to determine whether demographic factors and patient medication history could predict adherence to psychotropic medication among patients in Oman. The assumptions of linear regression—such as linearity, normality, independence, and homoscedasticity—were evaluated. The results showed no initial signs of multicollinearity. Subsequent tests confirmed the absence of multicollinearity [mean variance inflation factor (VIF) = 3], and the residuals were found to be normally distributed.

### 2.6. Ethical considerations

The Research and Ethics Committee at Al Masarra Hospital granted ethical approval for this study, and The Ministry of Health Research and Ethics Committee (Reference: MH/DGHS/DPT/705/2020) approved this study. Survey data cannot be publicly archived. Participants were assured of anonymity and informed that their participation was entirely voluntary, with the option to withdraw at any time after providing informed consent. Participants were identified using ID numbers to ensure that no names were recorded. It was made clear that participation would not affect their treatment plans, regardless of whether they chose to participate or withdraw from the study.

## 3. RESULTS

The authors of this study distributed 200 questionnaires, of which 175 were completed and returned, resulting in a response rate of 87.5%. After data cleaning and the removal of the incomplete questionnaires, 151 eligible participants were identified. Analysis of the 151 respondents revealed that the majority were aged between 26 and 45 years (59.6%, *n* = 90), with a gender distribution of 58.9% male (*n* = 89) and 41.1% female (*n* = 62). A significant proportion of the participants were either married (47.7%, *n* = 72) or single (42.4%, *n* = 64). Additionally, 64.2% (*n* = 97) had completed higher education, while only 3.3% (*n* = 5) reported having no formal education. Employment status was relatively balanced, with 45.0% (*n* = 68) employed and 55.0% (*n* = 83) unemployed. Furthermore, 23.8% (*n* = 36) reported having a chronic illness, and the same proportion indicated substance use. These demographic and health-related characteristics are summarized in Table 1.

Table 2 provides detailed information on participants’ mental health conditions and treatment regimens; in terms of specific psychiatric diagnoses, 45.0% of respondents had schizophrenia, 15.2% had depression, 16.6% had bipolar disorder, and 10.6% had anxiety. A substantial measure of insight into their illness was reported, with 68.9% of participants acknowledging their mental health condition. Treatment satisfaction levels were high, with 84.1% of participants expressing contentment with their treatment regimens. However, knowledge about medication among participants was a concern, with only 16.6% reported being informed about their medication, while 83.4% lacked sufficient understanding.

The composite score for each tool was calculated by summing the participants’ responses from the questionnaire. The average medication adherence score was 5.71 (SD = 2.45). Participants were classified into three groups based on their medication adherence levels: low, moderate, and high. This classification uses a method that calculates the grade range by subtracting the lowest score from the highest score and then dividing the result by three. Based on this method, the adherence scores were categorized into three groups: low (0–3 points), moderate (4–7 points), and high (8–10 points). The study findings indicated that most participants had a moderate level of medication adherence (49.67%, *n* = 75), while high adherence was reported in 28.48% (*n* = 43) and low adherence in 21.85% (*n* = 33).

The model demonstrated a moderate positive correlation, with an R-value of 0.593 and an R-squared value of 0.352, indicating that approximately 35.2% of the variance in the adherence levels is explained by these diverse factors. The analysis revealed that marital status had a statistically significant negative impact on medication adherence, with a coefficient of −0.886 (*p* = 0.001). Increased insight into one’s illness correlated with poorer medication adherence, as indicated by a significant coefficient of −2.082 (*p* = 0.000). Coefficient analysis revealed a negative influence of treatment satisfaction on adherence levels, with a coefficient of −2.177 (*p* = 0.000). Factors such as age group and gender did not show significant effects, with p-values of 0.766 and 0.458, respectively. Other variables, such as knowledge about medication, yielded a coefficient of 0.301; however, they were not statistically significant (*p* = 0.528). The analysis, supported by a model regression sum of squares of 316.628 and a residual mean square of 4.191, highlights the complex nature of medication adherence in mental healthcare (Table 3).

## 4. DISCUSSION

The findings of this study indicate that the average medication adherence score among participants was 5.71 (SD = 2.45), reflecting a moderate level of adherence. This score aligns with existing literature on adherence in individuals with mental health disorders, suggesting that while some patients adhere to their treatment, many do not achieve optimal adherence.^22^ Notably, 49.7% of participants exhibited a moderate level of adherence, a finding particularly relevant within the Omani cultural and healthcare context.^23^ This moderate level of adherence likely reflects a combination of willingness to seek treatment and persistent barriers such as cultural stigma, personal beliefs about mental illness, and limited understanding of psychiatric conditions.^23^ Although 68.9% of participants reported having insight into their illness—a factor generally associated with better adherence—this did not consistently translate into higher compliance, highlighting the complexity of this relationship.

Mental health literacy was notably low, with only 16.6% of participants reporting adequate knowledge about their medication—highlighting significant gaps in patient education and clinician communication. Despite this, 84.1% of participants expressed satisfaction with their treatment regimens, highlighting the potential of positive clinical experiences to support adherence. Enhancing patient education within a supportive healthcare environment may empower individuals to engage more actively with their treatment.^17^ The interaction between treatment satisfaction, insight, and improved education may help bridge the gap between treatment intent and adherence within Oman’s socio-cultural context.

The linear regression analysis identified key factors influencing medication adherence among psychiatric patients in Oman, highlighting both the challenges faced and potential avenues for intervention. Marital status emerged as a significant predictor of adherence, with unmarried individuals exhibiting lower adherence levels—a trend supported by existing literature. For example, a study conducted in Nigeria found that married patients with schizophrenia were significantly more adherent to their medication regimens, attributing this to increased family support and supervision.^18^ Similarly, research from Iran reported that married individuals with bipolar disorder exhibited higher treatment adherence, likely due to emotional and logistical support from their spouses.^24^ These findings align with the cultural context of Oman, where family structures are deeply embedded in daily life and often serve as a primary source of emotional and practical support. Involving families in psychoeducation and treatment planning has been shown to improve adherence outcomes across diverse settings,^19^ highlighting the importance of culturally sensitive, family-inclusive approaches in mental healthcare delivery in Oman.

Paradoxically, greater insight into one’s illness was associated with *lower* medication adherence in our Omani cohort—a pattern documented in recent research. Studies suggest that when insight coexists with internalized stigma, it can increase emotional distress and paradoxically decrease adherence.^20^ Notably, an Arab sample from Egypt revealed that higher insight and stronger spiritual beliefs were associated with better adherence. In contrast, concerns about medication had an adverse effect.^21^ These mixed findings suggest that the cultural context—particularly the intensity of stigma—modulates whether insight fosters or impedes adherence.

Additionally, the positive association between treatment satisfaction and adherence in our study aligns with meta-analytic findings identifying satisfaction as a robust predictor of adherence, particularly through stronger patient–provider relationships.^25^ Recent evidence from Middle Eastern settings highlights that stigma—particularly among healthcare providers and the public—remains a significant barrier to effective treatment engagement and service satisfaction.^26,27^ Thus, both insight and satisfaction interact with the level of stigma embedded in the social and clinical environment, influencing adherence outcomes in Oman.

## 5. IMPLICATIONS

The findings highlight the necessity for a comprehensive, culturally sensitive approach to mental healthcare in Oman to enhance medication adherence. Enhancing mental health literacy through targeted educational initiatives for patients, families, and communities can help dispel misconceptions about psychiatric disorders and emphasize the importance of adherence.^28,29^ As public awareness increases, treatment methods should be aligned with local traditions and social norms to foster a supportive environment for those seeking care.^30^ Prioritizing patient-centred care and enhancing provider–patient communication can strengthen therapeutic alliances and encourage active participation in treatment decisions.^31^

Anti-stigma campaigns are also crucial for reducing barriers to care and improving adherence to prescribed treatments.^32^ To further support adherence, integrated services that address both psychiatric needs and social determinants of health—such as housing, employment, and social support—are essential.^33^ Interventions such as counseling, peer support groups, and access to community resources can help overcome challenges that hinder treatment compliance.^34^ Policymakers should use these findings to guide resource allocation and strengthen the mental healthcare infrastructure.^35^ Establishing ongoing monitoring and evaluation mechanisms is vital for assessing adherence trends and evaluating the effectiveness of implemented strategies.^36,37^ These measures will contribute to a more holistic and sustainable mental health system in Oman, improving patient outcomes and system resilience

## 6. LIMITATIONS AND RECOMMENDATIONS

This study has several limitations. Its cross-sectional design prevents causal inferences, and the relatively small, single-centre sample restricts generalizability. Moreover, the reliance on self-reported data may introduce reporting bias. Key confounding variables, such as socio-economic status and family support, were not thoroughly examined, and the sample lacked demographic diversity.

Future research should adopt a longitudinal design with a larger, multi-site sample to improve generalizability. Employing mixed-method approaches that integrate both quantitative and qualitative data would provide a more comprehensive understanding of medication adherence. Moreover, future studies should systematically assess potential confounders to clarify their impact on adherence behaviors among individuals with psychiatric disorders.

## 7. CONCLUSION

In summary, medication adherence among psychiatric patients in Oman is moderate, with unmarried status and greater insight into illness linked to lower adherence. At the same time, higher treatment satisfaction is associated with better adherence. Addressing these factors through culturally sensitive education and support strategies is essential for enhancing adherence and improving treatment outcomes.

## DATA AVAILABILITY

Data available upon request.

## AUTHORS’ CONTRIBUTIONS

Study design: MQ, SAH, MAMA. Data collection: MAB, MAM, MABR. Data analysis: MQ, SAH, MAB, MAM. Study supervision: MQ, SAH, MAB, KAR, MABR. Manuscript writing: MQ, SAH, MAB, KAR. Critical revisions for important intellectual content: MQ, SAH, MAM, KAR, MABR.

## ACKNOWLEDGMENTs

The author expresses gratitude to all individual who contributed substantially to this study. Their collaboration and assistance were vital to the successful completion of this project.

## COMPETING INTERESTS

The authors have no conflicts of interest to declare.

## Figures and Tables

**Table 1.** Participants demographics.

**Age (years)**		**Education**	
18–25	27 (17.9%)	School-based	49 (32.4%)
26–45	90 (59.6%)	Higher education	97 (64.2%)
46–60	34 (22.5%)	Not educated	5 (3.3%)
**Gender**		**Employment**	
Male	89 (58.9%)	Employed	68 (45.0%)
Female	62 (41.1%)	Not employed	83 (55.0%)
**Marital status**		**Chronic illness**	
Single	64 (42.4%)	Yes	36 (23.8%)
Married	72 (47.7%)	No	115 (76.2%)
Divorced	9 (6.0%)	**Substance use**	
Widow	6 (4.0%)	Yes	36 (23.8%)
		No	115 (76.2%)

**Table 2.** Psychiatric and medication history.

**Diagnosis**		**Insight into illness**	
Schizophrenia	68 (45.0%)	Yes	104 (68.9%)
Depression	23 (15.2%)	No	47 (31.1%)
Bipolar	25 (16.6%)	**Treatment satisfaction**	
Anxiety	16 (10.6%)	Yes	127 (84.1%)
Other	19 (12.6%)	No	24 (15.9%)
**Previous admission**		**Knowledge about medication**	
Yes	78 (51.7%)	Yes	25 (16.6%)
No	73 (48.3%)	No	126 (83.4%)

**Table 3.** Model summary of linear regression.

**Model**	***R***	***R*- square**	**Adjusted *R*-square**	**Std. error of the estimate**	**Change statistics**				
					***R*-square change**	***F* change**	**df1**	**df2**	**Sig. *F* change**
1	0.593	0.352	0.301	2.047	0.352	6.868	11	139	0.000
**Model**	**Unstandardized coefficients**		**Standardized coefficients**	**t**	**Sig.**	**95% Confidence interval for B**			
	**B**	**Std. error**	**Beta**			**Lower bound**		**Upper bound**	
(Constant)	10.419	2.015		5.171	0.000	6.436		14.403	
Age group	0.094	0.314	0.024	0.298	0.766	−0.527		0.714	
The gender of the patient	0.305	0.410	0.062	0.744	0.458	−0.506		1.117	
Marital status	−0.886	0.264	−0.272	−3.352	0.001	−1.409		−0.363	
Educational level	0.060	0.196	0.026	0.309	0.758	−0.327		0.448	
Diagnosis	−0.031	0.120	−0.018	−0.261	0.794	−0.268		0.205	
H/O previous admissions	0.628	0.388	0.129	1.621	0.107	−0.138		1.395	
Knowledge about medication	0.301	0.475	0.046	0.633	0.528	−0.638		1.240	
Comorbid history	0.085	0.450	0.015	0.188	0.851	−0.805		0.974	
Substance use	−0.164	0.442	−0.029	−0.371	0.711	−1.038		0.710	
Insight	−2.082	0.426	−0.395	−4.885	0.000	−2.925		−1.240	
Treatment satisfaction	−2.177	0.500	−0.326	−4.355	0.000	−3.166		−1.189	

Predictors: (Constant) treatment satisfaction, educational level, diagnosis, substance use, history of (H/O) previous admissions, marital status, knowledge about medication, comorbid history, insight, age group, and gender of the patient.
